# Education Research: Feasibility and Impact of Academic Half-Day at a Large Academic Neurology Residency Program

**DOI:** 10.1212/NE9.0000000000200294

**Published:** 2026-02-10

**Authors:** Julia H. Greenberg, Riddhi Patel, Thomas A. Flagiello, Sungita Kumar, Nisha Aparna Malhotra, Nithisha Prasad, Alexandra Kvernland, Robert W. Charlson, Rajeev Motiwala, Ariane Lewis, Arielle M. Kurzweil

**Affiliations:** 1 Neurology, Stanford University, CA;; 2 Neurology, NYU Langone, New York, NY; and; 3 Neurology, Columbia University, New York, NY.

## Abstract

**Background and Objectives:**

The aim of this study was to assess the limitations of a traditional twice-daily lecture format and evaluate the feasibility and impact of implementing an academic half-day (AHD) for neurology residents at a multisite academic institution. AHD has the potential to improve attendance, satisfaction, and clinical competency compared with traditional didactics in graduate medical education. However, its feasibility and impact within neurology residency programs remain underexplored, with few adopting this model to date. Coverage logistics, faculty availability, and neurologic emergencies continue to pose challenges, particularly in large, multisite institutions.

**Methods:**

A needs assessment survey was administered to 36 neurology residents (postgraduate year [PGY]2–PGY4) in spring 2024 to evaluate attendance, satisfaction, and suggestions for improvement. Attendance was recorded over 1 month (August 2024). Based on survey feedback, a new 3.5-hour AHD curriculum was developed collaboratively by residents and faculty, held Tuesday mornings every week with varied lecture formats. One resident per class covered urgent clinical duties at each of 3 sites, while faculty and advanced practice providers (APPs) independently conducted rounds. Attendance was recorded for one month after implementation (September 2024), and surveys were distributed to assess satisfaction with both the curriculum and the coverage model. Residency In-Training Examination (RITE) scores were compared between the 2022 and 2024 cohorts (before AHD) and the 2025 cohort (after AHD implementation).

**Results:**

The needs assessment (n = 27) identified major barriers, including prerounding (78%), urgent clinical situations (37%), and Epic messaging during lectures (30%). Pre-AHD attendance averaged 35% (n = 36), which improved to 79% after AHD (*p* = 0.006). Residents reported greater engagement, concentration, retention, and camaraderie. Average RITE scores improved for the 2025 cohort compared with previous years (71% vs 65%, *p* = 0.0013). Faculty and APPs reported positive or neutral effects on workflow (82.6% and 100%, respectively) and patient safety (95.6% and 100%, respectively).

**Discussion:**

Implementation of AHD across a large, multisite neurology residency program was feasible and associated with higher attendance, improved resident satisfaction, and enhanced RITE performance, without adverse effects on workflow or patient safety. Key factors for success included resident involvement, a targeted needs assessment, and strong coverage support from faculty and APPs.

## Introduction

The Accreditation Council for Graduate Medical Education mandates that postgraduate residency and fellowship programs implement a formal curriculum covering a range of core objectives. However, it does not specify the frequency, modality, format, or length of instruction.^1^ Most neurology residency programs meet these educational requirements through traditional daily didactic sessions.^1^ However, duty hour restrictions, clinical responsibilities, and increasing documentation burden have contributed to declining resident attendance and engagement.^1-3^ In addition, evidence regarding the efficacy of traditional daily didactics on resident satisfaction, knowledge, and clinical performance remains limited and inconsistent.^1,2^

Academic half-day (AHD) is an alternative educational format that provides a regularly scheduled, protected block of time (typically 2–3 hours) dedicated to resident education. Ideally, trainees are relieved of patient care responsibilities during this time.^2^ AHD integrates cognitive load theory, which highlights how simultaneous clinical duties and didactics can overload residents' learning capacity, as well as principles of Kolb's experiential learning theory and self-directed/self-regulated learning, which support taking ownership of one's learning through reflection, deliberate practice, and knowledge application.^4^ This format has gained increasing popularity among residency programs across various specialties in the setting of increasing clinical responsibilities limiting attendance, reduced engagement over virtual platforms, and time constraints that hinder effective longitudinal learning.^1^ In internal medicine residency programs, studies at Cambridge Health Alliance (a public community hospital) and University of Cincinnati (an academic health center) have demonstrated improved attendance, resident satisfaction, and clinical competency with AHD compared with traditional noon conferences.^2^

Despite these promising findings, limited evidence exists regarding the feasibility and success of AHD within neurology residency programs—particularly at large, urban academic centers in the United States. A survey of Canadian neurology program directors reported widespread AHD adoption with high resident attendance but emphasized the need for further research into its educational value in neurology specifically.^5^ Implementing AHD in a neurology program poses notable logistical challenges. These include ensuring a unified learning environment across sites, maintaining adequate clinical coverage without compromising patient care, and securing faculty and space availability. One of the most significant barriers to AHD adoption is developing a safe and effective coverage model that accommodates resident education while preserving clinical care. A qualitative study from the Department of Pediatrics at Children's Hospital Colorado highlighted emotional strain, technological barriers, and patient safety concerns experienced by attendings when tasked with providing coverage in the absence of trainees.^6^ These challenges may be further magnified in large academic institutions with high patient volumes and quality metrics, such as timely consult completion. One study found AHD implementation to be less common in academic centers (30% vs 71.4%), in the Northeast (14% vs 52.2%), and among programs with larger residency classes.^7,8^ In neurology specifically, frequent and time-sensitive emergencies—such as stroke codes, spinal cord compression, brain hemorrhages, and status epilepticus—further complicate AHD implementation.

Our neurology residency program is based in Manhattan. The program includes 36 residents. We are responsible for covering 3 large hospital systems in a 10-block radius, which includes a Level 1 trauma center and 2 comprehensive stroke centers. After performing a needs assessment regarding how to optimize resident education, we transitioned from the traditional format of twice-daily lectures to AHD. In this study, we (1) discuss the challenges of the traditional lecture format at our large academic neurology program; (2) present the impact of a resident-initiated AHD curriculum on resident attendance, satisfaction, and in-service test performance; and (3) outline the coverage model across a multisite program to facilitate AHD.

## Methods

### Needs Assessment Survey

In the spring of 2024, the education chief residents (PGY-4) designed a needs assessment survey to identify barriers to didactic attendance and assess satisfaction with the current curriculum. The survey also aimed to gauge resident preferences between the traditional twice-daily lecture format and AHD. After review by the PD and associate program directors, the survey was distributed through email to all PGY-2 through PGY-4 neurology residents (n = 36). Residents were reminded twice in person to complete the anonymous survey. The survey included 9 open-ended questions and 1 multiple-choice question (eAppendix 1). The open-ended items asked about barriers to attendance, strengths and weaknesses of the current curriculum, and suggestions for improvement. The multiple-choice item asked whether residents preferred the traditional format or the AHD model. Multiple-choice responses were analyzed quantitatively. Open-ended responses were thematically grouped based on common language and recurring themes by the study authors.

### AHD Structure and Curriculum

To address gaps identified in the needs assessment survey, education chief residents and education faculty designed a new AHD-based curriculum to permanently replace the traditional twice-daily lecture format, beginning in September 2024. The AHD curriculum is centered around the following principles: (1) protected time and unified one-room schoolhouse in a multisite program; (2) chief resident involvement in curricular design and logistics; (3) shared coverage responsibility among PGY2-PGY4 residents, faculty, and APPs; (4) multimodal teaching formats—lectures, case-based learning, board review, hands-on workshops (EMG, Botox for migraines, vagus nerve stimulation training), and practice questions; (5) asynchronous learning with recordings uploaded for residents covering services, on vacation, or on night float rotations; and (6) continuous improvement models based on regular formal and informal feedback. These goals are inspired by internal informal discussions and suggestions provided by residents and faculty members, including the PD and associate PDs.

Tuesday mornings were selected for the 3.5-hour AHD based on several scheduling considerations: Monday care team transitions, continuity clinic sessions (which occur on Monday, Wednesday, and Thursday afternoons), the historical timing of grand rounds (Tuesday morning), and patterns of consult and inpatient workload (typically highest in the afternoons). The AHD curriculum was drafted by chief residents and reviewed by the education faculty. Sessions include grand rounds, case-based discussions, simulations, and traditional didactics (30 minutes), followed by 10–15 minutes of interactive board-style questions. Five-minute breaks are scheduled between lectures. A block-based format is used, with each subspecialty receiving 1–4 weeks of focus based on educational breadth to ensure a comprehensive and structured curriculum. Block duration was determined using chief resident experience, survey feedback, historical Residency In-Training Examination (RITE) scores, and feedback from a designated faculty member within each subspecialty of neurology (Table 1). In addition to AHD, we retained 2 weekly case-based conferences (Wednesdays and Thursdays, 8–9 a.m.) because of high attendance and strong faculty involvement. To accommodate scheduling realities—including night float, vacation, and interviews—AHD sessions are recorded and made available for asynchronous learning. However, to promote in-person engagement, no live remote link is offered.

**Table 1:** Design and Logistics of Academic Half-Day (AHD)

Clinical setting	Three sites, urban large academic neurology resident program
Program size	Thirty-nine residents (35 adult neurology, 2 pediatric neurology, 2 neuropsychiatry)
AHD leadership	Five chief PGY-4 chief residents, program director (PD), and 2 associate program directors (APDs)
AHD schedule	Tuesdays, 8:00–11:30 am *Grand rounds 8:00–9:00 am
Residents exempt from attendance	Vacation, night float, covering residents (1 PGY-3, 1 PGY-4 ± 1 PGY-2)
Curriculum structure	Block-based curriculum: Neuromuscular 4 wk, Neuro-Ophthalmology 2 wk, Neuro-immunology 4 wk, Neuro-otology 1 wk, Neuro-ICU 1 wk, Headache 2 wk, Sleep 1 wk, Neuro-oncology 2 wk, Epilepsy 4 wk, Movement disorder 3 wk, Vascular neurology 4 wk, Cognitive 2 wk, Neuro-ID 1 wk
Lecture formats	1. Grand rounds 2. Traditional 30-min didactics followed by 10 min of board-style questions 3. Simulations/interactive sessions (e.g., EMG demonstration and Botox demonstration/practice) 4. Case-based lectures 5. Board-style question review
Lecture duration	40 min
Lecturers	Inpatient and outpatient clinicians across all subspecialties Education chief residents
Additional weekly lectures not included in AHD	Morning report: Wednesdays 8:00–9:00 am Case-based patient presentation by PGY-2/3 of a classic neurologic condition, followed by attending lecture on the topic Professor rounds: Thursdays, 8:00–9:00 am Case-based presentation(s) of complex/mystery condition, insights provided by various faculty members

### Coverage Model

The clinical coverage model during AHD is provided in Table 2. Two residents (1 PGY-3 and 1 PGY-4) are assigned to cover urgent consults and stroke codes across 2 of the 3 hospital sites. Attendings and advanced practice providers (APPs) continue managing routine rounding and urgent care needs. The PGY-2 resident in the Neuro-intensive care unity (ICU) (at 1 site only) attends AHD if there are no active emergencies but is expected to leave if clinical issues arise. Additional support is provided by rotating medical students and off-service learners (e.g., psychiatry residents), who assist with patient care during the AHD window. At the third, lower volume site, a faculty member independently manages clinical issues during AHD. This coverage structure results in an average of 0–1 missed AHD sessions for PGY-2 residents and approximately 5 sessions for each PGY-3 and PGY-4 resident (excluding absences due to vacation, night float, or interviews, which are unrelated to the didactic format).

**Table 2:** Coverage Models for Each of the 3 Sites During Academic Half-Days (AHDs) Compared With Regular Clinical Days

Hospital 1	AHD coverage	Regular coverage
1. General inpatient team	1 attending, 1 APP, 1–2 medical students	1 attending, 1 PGY-2, 1 PGY-3, 1 APP, 1–2 medical students
1. General consult team	1 attending, 1–2 medical students ^a^1 PGY-3	1 attending, 1 PGY-2, 1 PGY-3, 1–2 medical students
1. Stroke team (inpatient + consults)	1 attending, 1 APPs, 1–2 medical students ^a^1 PGY-3	1 attending, 1 PGY-2, 1 PGY-4, 1 APP, 1–2 medical students
4. Neurocritical care team	1 attending, 1 PGY-2	1 attending

Hospital 2	AHD coverage	Regular coverage
1. Stroke inpatient team	1 attending, 1–2 APPs, 1–2 medical students	1 attending, 1 PGY-2, 1 PGY-3/4, 1–2 APPs, 1–2 medical students
1. Stroke consult team	1 attending ± 1 stroke fellow 1 PGY-4	1 attending, 1 PGY-3, ± 1 stroke fellow
1. Neurocritical care consult	1 attending ^a^1 PGY-4 Rounds in the afternoon after AHD	1 attending, 1 PGY-3/4
1. Neurology general consult team	1 attending, 1 psychiatry PGY-1, 1–2 medical students ^a^1 PGY-4	1 attending, 1 PGY-2, 1 PGY-4, 1 psychiatry PGY-1, 1–2 medical students

Hospital 3	AHD coverage	Regular coverage
1. Neurology consult team	Attending holds pager Rounds with residents and attending in the afternoon after AHD	1 attending, 1 PGY-3, 1 PGY-2, 1 psychiatry PGY-1

a Resident covers all stroke codes and all urgent consults (stroke and general neurology).

### Outcome Measures

Primary outcome measures included (1) average lecture attendance before and after implementation of AHD; (2) qualitative and quantitative resident satisfaction with the curriculum; (3) average RITE scores before and after implementation of AHD; and (4) faculty, APP, and resident satisfaction with the coverage system.

Resident satisfaction with the new curriculum and coverage model was assessed through a postimplementation survey administered 6 months after AHD began in March 2025, 1 month after the RITE (eAppendix 2). The cross-sectional survey consisted of 11 multiple-choice questions and 1 open-ended response. It was distributed through email to all PGY-2 through PGY-4 neurology residents (n = 36).

Lecture attendance was tracked using QR code sign-ins for 1 month before AHD implementation (August 2024) and 1 month after implementation (September 2024). Attendance during both periods was compared using the Mann-Whitney *U* test in IBM SPSS Statistics software version 29. Resident satisfaction with the curriculum and coverage system was assessed through a Google Form survey consisting of 11 multiple-choice questions and 1 open-ended question, administered 6 months after implementation of AHD (eAppendix 2). The survey assessed the percentage of lectures attended (<25%, 25%–50%, 50%–75%, or >75%) and perceptions of AHD's impact on workload, quality of learning, patient care, attention span, and overall comparison with the traditional twice-daily format. The response scale varied per question. Answers to the open-ended question about any additional feedback regarding AHD were categorized qualitatively into common themes by the author.

Faculty (n = 23 respondents of 60 rotating on inpatient services) and APP (n = 7 respondents of 9) satisfaction with the AHD coverage system was assessed using a survey, approximately 6 months after implementation (eAppendix 3). This survey included 4 multiple-choice questions and 1 open-ended question and was also distributed through email. Topics included respondent roles, impact of AHD on workflow, patient care and safety, and resident performance and knowledge. Multiple-choice responses generally offered 3 categories: positive, neutral, or negative. Open-ended responses were reviewed and categorized by the theme.

**Figure 1:**
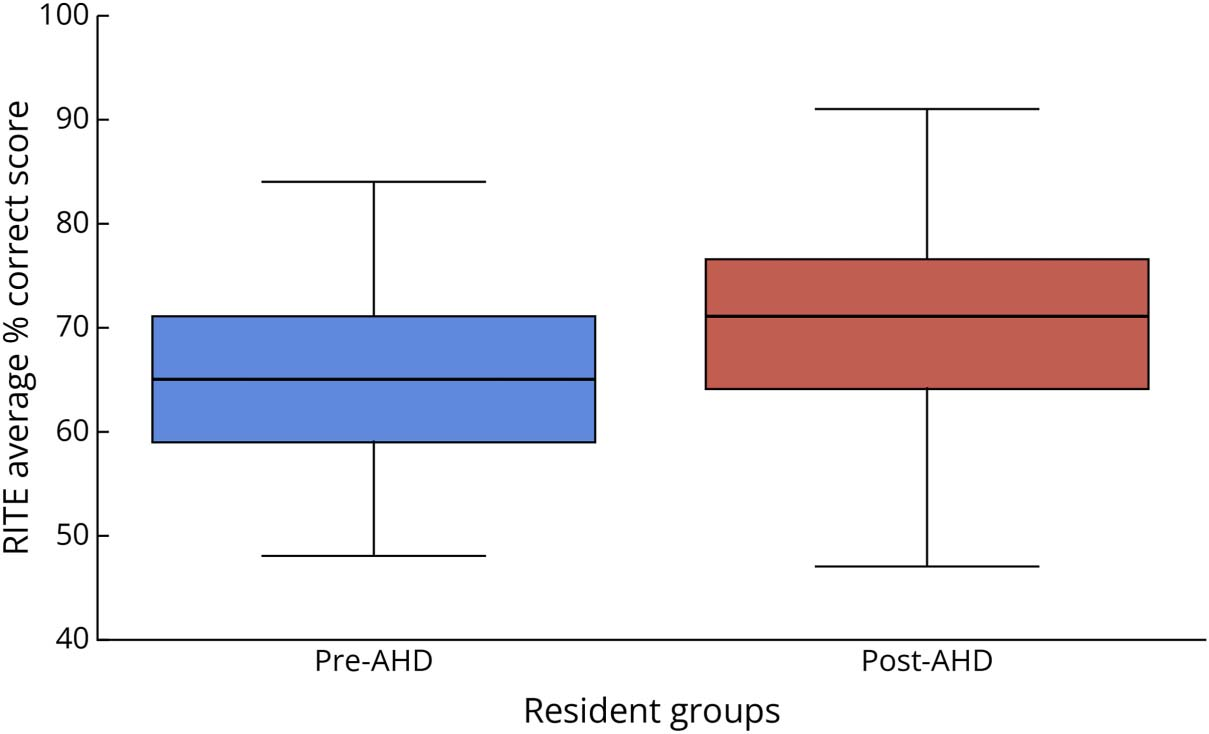
A Univariate Analysis Using the Mann-Whitney *U* Test in SPSS Software Compared RITE Scores of All PGY Classes Over the 3 Academic Years Preceding Implementation of AHD to RITE Scores of All PGY Classes During the Academic Year After Implementation of AHD *p* value = 0.0013 (*p* < 0.05 = statistically significant). AHD = advanced practice provider; RITE = Residency In-Training Examination.

RITE scores (total % correct) from 2022 to 2025 were anonymized by administrative staff and provided for analysis. Scores from 2022 to 2024 were grouped as the pre-AHD implementation cohort, whereas scores from 2025 were used as post-AHD data. All 3 classes (PGY-2 through PGY-4) were included in both cohorts. To account for the non-normal distribution of data, we used the Mann-Whitney *U* test in IBM SPSS Statistics software version 29 to compare RITE score distributions between the 2 independent groups. This nonparametric analysis allowed us to determine whether a statistically significant difference existed between median scores in the pre-AHD and post-AHD periods (Table 3 and Figure 1). Combining 3 years of pre-AHD data helped minimize confounders such as the COVID-19 pandemic, class size variation, examination difficulty, and individual variability, because 2 classes were present in both cohorts. According to the American Academy of Neurology data provided to program leadership across the country, national average RITE scores were 66% in 2022, 60% in 2023, and 66% in 2024 and 2025.

**Table 3:** Comparison of RITE Scores Before AHD Implementation (2022–2024) and After AHD Implementation (2025) Using the Mann-Whitney *U* Test Analysis

Group	Sample	Mean	Std	Min	25%	50%	75%	Max	*U* statistic	*p* Value
After AHD	36	70.8	9.38	47.0	64.0	71.0	76.5	91.0	1,111.5	0.0013
Before AHD	97	64.96	8.41	48.0	59.0	65.0	71.0	84.0		

Abbreviations: AHD = advanced practice provider; RITE = Residency In-Training Examination.

The Mann-Whitney *U* test is used in SPSS software for non-normally distributed data to compare medians of the 2 cohorts, pre-AHD and post-AHD. The post-AHD cohort includes PGY-2 through PGY-4 RITE scores (% total correct) from 2025, and the pre-AHD cohort includes PGY-2 through PGY-4 RITE scores from 2022 to 2024. *p* Value ≈0.0013 (*p* < 0.05 = statistical significance).

### Standard Protocol Approvals, Registrations, and Participant Consents

The institutional review board (IRB) at our medical center does not review educational quality improvement initiatives for approval or exemption because they do not consider these initiatives and related data to be human subjects research. The IRB defines research as “a systematic investigation, including research development, testing, and evaluation, designed to develop or contribute to generalizable knowledge.”^9^ As such, IRB review was waived because the implementation of AHD was conducted as an internal educational quality improvement initiative rather than research intended to generate generalizable knowledge. All data were collected anonymously.

### Data Availability

Anonymized data not included in this article can be provided on request to qualified investigators.

## Results

### Twice-Daily Lectures: Attendance and Feedback

Average lecture attendance during the 1-month period before AHD was 35% but varied by session type and time of day. Attendance on average was highest for morning reports (71%, 25.5/36), followed by grand rounds (65%, 23.5/36) and case-based lectures (50%, 18/36).

**Figure 2:**
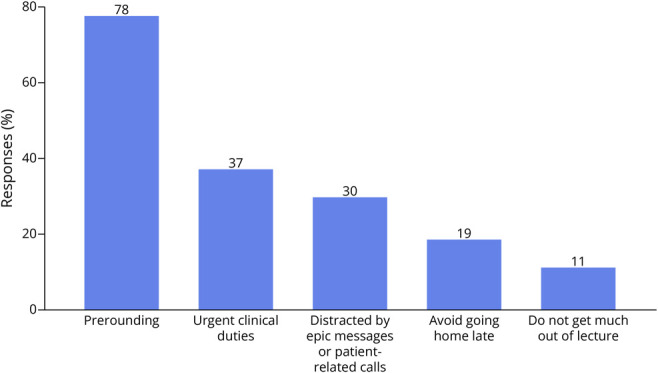
Common Barriers to Twice-Daily Didactics' Attendance (n = 27 Respondents)

The needs assessment survey was completed by 75% (27/36) of neurology residents in full (100% [12/12] PGY2, 66.6% [8/12] PGY3, 25% [3/12] PGY4, 5 unlabeled respondents). The most cited barriers to attendance included competing prerounding obligations (78%, 21/27), fulfilling emergency clinical responsibilities (37%, 10/27), and receiving EPIC messages during lectures (30%, 8/27, Figure 2). On the multiple-choice question, 88.5% of respondents were in favor of switching to the AHD format. Thematic analysis of open-ended comments revealed several consistent themes: a need for protected educational time, a desire for varied lecture formats, and an appreciation for in-person learning to foster both education and camaraderie. Residents also emphasized the importance of a structured and dependable coverage system during AHD. The most frequently expressed concerns about AHD among residents included maintaining concentration over a 3.5-hour span and needing to stay late to complete clinical work deferred during AHD.

### AHD: Attendance and Feedback

**Figure 3:**
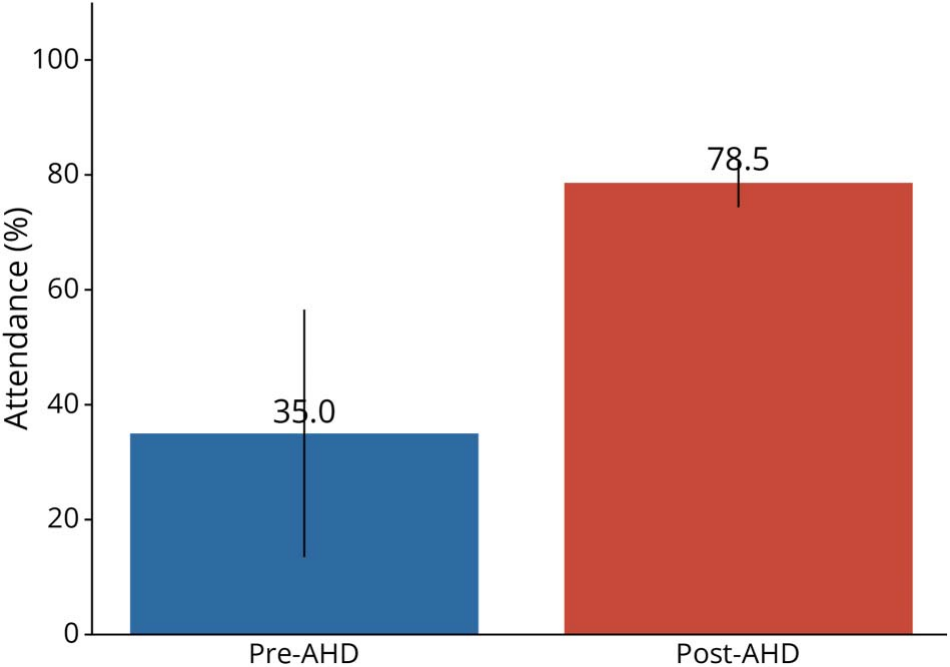
Comparison of Average Lecture Attendance Rates Using a Mann-Whitney *U* Test for Pre-AHD (n = 10 Lectures) and Post-AHD (n = 4 Lectures) Scores Over a 1-Month Period for 36 Residents *p* value = 0.006 (*p* < 0.05 = statistically significant). AHD = academic half-day.

After AHD implementation, average monthly attendance increased from 35% to 79% and was found to be statistically significant using the Mann-Whitney *U* test (*p* = 0.006) (Figure 3). Among the 25 residents who responded to the 6-month post-AHD survey (58.3% of PGY-2s, 50% of PGY-3s, 83.3% of PGY-4s, and 2 anonymous-year responses), 92% preferred the AHD format over traditional didactics. In addition, 96% reported that key barriers previously impeding attendance—such as lack of protected time and conflicting clinical duties—had improved with AHD implementation. The remaining 4% indicated that these barriers persisted. In the open-ended feedback section as well as during informal in-person feedback, most of the residents cited improved engagement, knowledge retention, and camaraderie as benefits of the AHD format.

### Faculty and APP Feedback After Implementation of AHD

**Figure 4:**
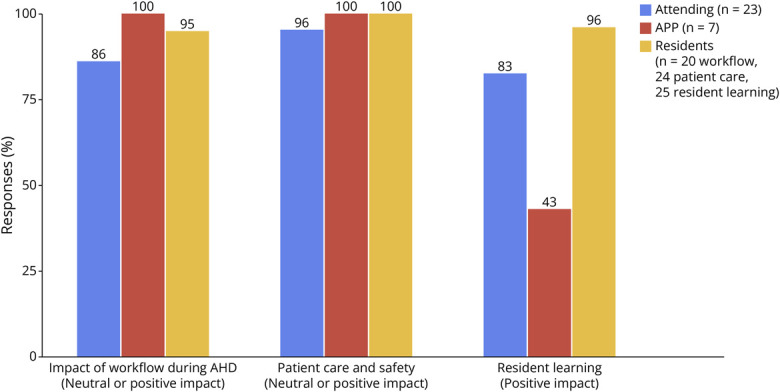
Satisfaction With Various Aspects of Coverage Model Across Residents, Faculty, and APPs (n = 52) APP = advanced practice provider.

Among the 30 respondents to the postimplementation feedback survey (23/60 faculty and 7/9 APPs), 100% of APPs reported a neutral or positive impact of AHD on workflow, patient care and safety, and resident learning (Figure 4). Among attending physicians, 82.6% indicated a neutral or positive impact on their workflow, 95.6% reported a neutral or positive effect on patient care and safety, and 82.6% reported a positive impact on resident learning. The remaining 17.4% either selected “neutral” or left the response blank.

On the open-ended section of the survey, commonly cited benefits by faculty included improved morale among residents, enhanced resident learning, more efficient rounds during AHD mornings, and the opportunity for APPs to take full ownership of patients. Concerns expressed by 2 faculty members included missed opportunities for bedside teaching during rounds 1 day per week. Three faculty and 2 APPs reported difficulty managing workload during high census days or when simultaneous stroke codes occurred. Some suggestions involved adding a second covering resident per site during days with a particularly high census, switching AHD to Tuesday afternoons after rounds, and establishing detailed contingency plans for emergency scenarios such as simultaneous stroke codes.

### RITE Score Comparison Between Traditional Twice-Daily Didactics and AHD

Program-wide RITE performance improved from a median score of 65% across all 3 classes (PGY-2 through PGY-4) during the 3 years before AHD implementation to a median score of 71% in the year after implementation. Using the Mann-Whitney *U* test, this difference was found to be statistically significant, with a *p* value of 0.0013 (Table 3 and Figure 1).

## Discussion

We present an AHD curriculum model that has proven successful at a large, northeastern academic program, despite previous studies suggesting that programs with similar characteristics are less likely to implement AHD effectively.^8^ Average lecture attendance increased from 35% to 79%, and qualitative survey feedback from residents was overwhelmingly positive, with many citing improvements in attendance, engagement, knowledge, and camaraderie/resident morale. In addition, average RITE scores improved significantly after the implementation of AHD.

Several aspects of our transition process contributed to the success of this model, including a program-specific needs assessment survey, active resident involvement in curriculum design and implementation, and strong coverage support from APPs and faculty.

The needs assessment survey helped identify key challenges with traditional twice-daily didactics, such as low resident attendance, limited participation, and reduced engagement and satisfaction. Grand rounds and case-based lectures were noted as the most attended and highly rated sessions, underscoring the demand for more diverse and interactive lecture styles. Transitioning to AHD provided a longer, uninterrupted block of educational time, enabling lectures to build on one another and incorporate demonstrations, simulations, and other interactive formats that were not feasible with the traditional 50-minute sessions. The AHD model promotes deeper, experiential learning by aligning with Kolb's 4 stages: concrete experience (case-based discussions, hands-on EMG, or neuro-otologic maneuvers), reflective observation (peer and instructor feedback), abstract conceptualization (linking theory to related topics such as sequential lectures on neuropathies), and active experimentation (applying knowledge in clinical vignettes).^4^

Chief residents offered valuable insights into the strengths and weaknesses of the curriculum in previous years, along with residents' logistical and academic needs, allowing for a more tailored and responsive design. This role also gave chiefs meaningful exposure to the administrative aspects of medical education—an excellent opportunity for those interested in academic leadership. As resident cohorts and educational needs evolve, there remains ongoing potential to enhance the curriculum by incorporating more interactive and creative sessions and introducing new topics. This will allow the AHD model to continue serving as a dynamic learning and leadership opportunity for future education chiefs.

Residents noted lack of protected time and competing clinical duties as major barriers to attending twice-daily didactics. This aligns with previous literature from non-neurology residency programs studying didactic format, which found that protected time was the primary driver of improved attendance.^1,8^ However, designing a satisfactory coverage system is difficult, particularly at large, multisite institutions, and previous studies have suggested that approximately 75% of programs require residents to be “somewhat responsible for pagers” during didactics.^8^ In addition, programs have run into issues with increased workload and dissatisfaction of faculty and APPs.^3^ Our coverage system enables protected education time without placing undue burden on covering residents, faculty, or APPs. Most participants reported satisfaction with the system, describing a neutral or even positive impact on workflow and noting no negative effects on patient care. A key factor in this success was the support of faculty and APPs covering inpatient services, along with the presence of rotating medical students and other rotators. This structure allowed for only 1 resident per class to be pulled for coverage, while maintaining a manageable workload for faculty.

Despite the curriculum's overall success, several areas remain for improvement. For instance, attention span was a concern among a few residents before the survey. This may be addressed by incorporating frequent breaks between lectures, offering a variety of teaching formats, and designing sessions to be more interactive. Although most faculty and APPs were satisfied with the coverage model, some expressed concerns about covering simultaneous emergencies, periods of high patient volume, and missing bedside teaching, particularly in the ICU. This model depends heavily on APP support and may not be generalizable to institutions with limited APP availability. However, it may be feasible to incorporate additional faculty support in those settings.

This study has several limitations. First, not all potential respondents completed the surveys, raising the possibility of sample bias, particularly as pertains to faculty feedback. Second, some residents may not have used QR codes to record attendance; however, there is no clear reason this would systematically differ between the 2 curricular models. Third, multiple curricular elements were changed simultaneously based on the needs assessment. These included fully protected time, the shift to more cohesive subspecialty blocks, and greater variation in lecture format, making it challenging to isolate the specific effect of the AHD format. This point is particularly relevant, given findings from a recent cross-sectional study published in *Neurology® Education*, which showed that improved attendance was associated with protected time, reduced didactic hours, and an increase in PD-led sessions—but not AHD alone.^8^ However, it is important to note that the AHD structure made the implementation of these curricular improvements feasible—enhancements that would have been difficult or impossible to achieve with a traditional twice-daily model. We did not study the impact of AHD on resident's well-being, burnout, or sense of community, but this would have been interesting. Residents informally subjectively endorsed improved camaraderie after the implementation of AHD. We also could not objectively compare satisfaction before and after AHD because we did not include the same quantitative questions on both surveys. We will consider further quantitative data collection and analysis in future work.

Finally, our RITE score analysis was based on only 1 year of postimplementation data, limiting our ability to assess long-term trends or cohort-specific variability. Furthermore, although standardized test scores are a valuable outcome, they may not fully capture resident competence when considered in isolation. While there are several factors that could have influenced RITE scores, the national average RITE scores for neurology residents were stable throughout this period (66% in 2022 and 2024 and 66% in 2025), while average scores in our program increased from 65% to 71%.

In summary, we share insights into a successful transition to an AHD curriculum at a large, academic, multisite neurology residency program. The AHD model led to improved resident attendance and higher RITE performance, with overall satisfaction reported across residents, faculty, and APPs. Future studies are warranted to evaluate the longitudinal impact of AHD, explore more comprehensive measures of resident performance as well as impact on resident well-being and burnout, and refine coverage systems for broader applications.

## Glossary

AHD: academic half-day
APP: advanced practice provider
IRB: institutional review board
PD: program director
RITE: Residency In-Training Examination
